# Spectrum of Omphalomesenteric Duct Related Anomalies and Their Surgical Management in Children

**DOI:** 10.7759/cureus.13898

**Published:** 2021-03-15

**Authors:** Muhammad Azhar, Naima Zamir, Syed R Taqvi, Mishraz Shaikh

**Affiliations:** 1 Paediatric Surgery, National Institute of Child Health, Karachi, PAK; 2 Paediatric Surgery, Jinnah Sindh Medical University, Karachi, PAK

**Keywords:** bleeding per rectum, umbilical anomalies in children

## Abstract

Objective

The aim of this study was to evaluate the clinical presentation and surgical management of omphalomesenteric duct (OMD) remnants in children.

Material and methods

A descriptive retrospective study was conducted at the Department of Paediatric Surgery of the National Institute of Child Health, Karachi, Pakistan, from April 2017 to January 2020. Children below 12 years of age with various OMD remnants were included in the study. Data regarding age of presentation, type of anomaly, and management collected during this period were reviewed and analyzed using SPSS Version 22 (IBM Corp., Armonk, NY, USA).

Results

A total of 86 patients, 47 males and 39 females, were managed during the study period. Intestinal obstruction was observed in 44 (51.16%) cases followed by OMD-related umbilical anomalies in 14 (16.27%) cases, acute abdominal pain in 12 (13.95%), rectal bleeding in 3 (3.48%) patients. In 13 (15.16%) cases, Meckel’s diverticulum was discovered incidentally. In 21 cases, wedge resection and ileal repair was performed, whereas 32 required segmental resection and end-to-end anastomosis, and in 32 cases ileostomy was created after resection. Histopathology showed the presence of ectopic mucosa in five cases.

Conclusion

Patients with OMD remnants had various presentations. The surgical procedure has to be tailored according to the clinical and surgical findings.

## Introduction

The omphalomesenteric duct (OMD), also called the vitelline or vitellointestinal duct (VID), is an embryonic structure providing communication from the yolk sac to the midgut during fetal development [1]. The natural fate of this structure is spontaneous obliteration, where it separates from the intestine during fifth and ninth weeks of gestation [2]. Failure of its regression results in a spectrum of anomalies including Meckel's diverticulum, patent OMD (POMD), Meckel’s band, omphalomesenteric cysts, umbilical sinus, or umbilical polyp [3]. The major burden of these malformations is encountered in the pediatric population when they present with complication caused by an underlying remnant. The intra-abdominal components of OMD remnants may remain asymptomatic or are incidentally discovered during a laparotomy for other reasons. However, those who become symptomatic can have a wide range of presentation based on the underlying anomaly. This include complications such as intestinal obstruction secondary to the band, intussusception, internal herniation, volvulus, or acute abdominal pain due to Meckel’s diverticulitis [4]. Anomalies related to the umbilicus present with umbilical discharge or polypoid growth protruding through the umbilicus [5]. Heterotrophic tissue is also known to be associated with OMD remnants, particularly with Meckel’s diverticulum. This predisposes to local hyperacidity and mucosal ulceration, causing recurrent abdominal pain or per rectal bleeding in children [6,7]. Various surgical options have been described in the literature, ranging from wedge or segmental resection in case of Meckel’s diverticulum to umbilical exploration in case of umbilical discharge. Most reports on symptomatic OMD focus on Meckel's diverticulum, whereas other related anomalies are given little attention. The aim of this study was to gather a single institutional experience on the various OMD remnants in children with an emphasis on the age and clinical presentation, intra-operative findings, surgical intervention performed, and the histopathological outcome.

## Materials and methods

A retrospective study of pediatric patients of both sex aged younger than 12 years with some form of OMD remnants treated at the Department of Paediatric Surgery, National Institute of Child Health, Karachi, Pakistan, from April 2017 to January 2020 was conducted. After approval from the Institutional Ethical Review board, medical records of patients with OMD remnants were reviewed to collect information regarding sex, age at presentation, type of anomaly identified, and surgical intervention performed. Patients were grouped as those presenting with acute abdomen (including intestinal obstruction and peritonitis), umbilical anomalies, per rectal bleeding, and incidental findings. Apart from baseline hematological work-up, patients with acute abdomen had X-rays/ultrasound of the abdomen, and wherever needed CT scan was also added. No radiological investigations were required for umbilical anomalies except those in doubt of internal communication with the gut, where a contrast study was conducted. Patients with intestinal obstruction or acute abdominal pain underwent laparotomy after initial resuscitation. The surgical procedure was tailored according to clinical condition of the patient and per-operative findings. In general, those with a narrow base Meckel’s diverticulum underwent wedge resection, while those with a wide base underwent segmental resection and anastomosis. However, in case of gross peritoneal cavity contamination, friable, and edematous gut, ileostomy was made irrespective of the anatomy of Meckel’s diverticulum. In patients with incidentally discovered Meckel’s diverticulum, no intervention was performed. However, in case where it was required due to primary surgical condition requiring diversion, site of Meckel’s diverticulum was chosen for ileostomy after its segmental resection. In cases of umbilical anomalies, polyps were excised under local anesthesia, whereas patients with the patent OMD underwent laparotomy. Medical records were also reviewed for histopathological results of excised tissue. All data were processed using descriptive statistical procedures for calculating means, standard deviations, frequencies, and percentages. Data were analyzed using SPSS Version 22 (IBM Corp., Armonk, NY, USA).

## Results

A total of 86 patients were evaluated during the study period, of which 47 (54.65%) were males and 39 (45.34%) were females, the age of patients ranged from 1 day to 12 years (mean: 4.83 ± 3.536 years). Table 1 provides the demographic data of the patients.

**Table 1:** Demographic characteristics

Clinical presentation	Age, years (means ± SD)	Sex (male/female)
Intestinal obstruction (n=44)	2.34±7.76	27/17
Abdominal pain (n=12)	4.12±6.03	5/7
Umbilical anomalies (n=14)	0.05±0.52	6/8
Rectal bleeding (n=3)	3.12±7.35	2/1
Incidental (n=13)	1.27±9.04	9/4

Among symptomatic patients, intestinal obstruction was the most common mode of presentation followed by umbilical anomalies and abdominal pain due to inflamed Meckel’s diverticulum. Table 2 provides a summary of clinical manifestation, type of anomaly, and surgical procedure performed.

**Table 2:** Summary of presentation, type of anomaly, and operation performed POMD, patent omphalomesenteric duct

Clinical presentation	Operative findings	No. of patients	Wedge resection and anastomosis	Segmental resection and anastomosis	Segmental resection and ileostomy	Total patients, N=86
Intestinal obstruction	Meckel's diverticulum with band	31 (70.45%)	17	8	6	44 (51.16%)
	Intussusception	09 (20.45%)	-	6	3	
	Internal herniation	03 (6.81%)	-	2	1	
	Volvulus	01 (2.27%)	-	-	1	
Abdominal pain	Diverticulitis	09 (76.47%)	2	7	-	12 (13.95%)
	Perforated Meckel’s diverticulum	03 (23.42%)	-	-	3	
Umbilical anomalies	POMD	06 (31.57%)	2	6	-	14 (16.27%)
	Prolapsed POMD	04 (21.05%)	-	2	-	
	Umbilical sinus	02 (10.52%)	-	-	2	
	Umbilical polyp	02 (15.78%)	-	-	-	
Rectal bleeding	Meckel’s diverticulum	3	-	3	-	3 (3.48%)
Incidental	Meckel’s diverticulum	13	-	1	7	13 (15.16%)

Mesodiverticular bands as a cause of intestinal obstruction due to internal herniation were seen in three patients. Fibrous band arising from the tip of Meckel’s diverticulum and attached to the umbilicus was observed in one case, which led to volvulus requiring resection of about 40 cm of the distal ileum. Three patients presented with per rectal bleeding. Among them, one patient underwent emergency laparotomy due to a high index of suspicion for intussusception; intra-operatively, it proved to be a bleeding Meckel’s diverticulum. Two other cases presented with a history of recurrent abdominal pain and passing melena. The abdominal CT scan showed a thickened tubular structure entrapped between the bowel loops. Diagnostic laparoscopy was performed, which revealed Meckel’s diverticulum that underwent segmental resection. Among patients with incidentally discovered asymptomatic Meckel’s diverticulum, there were four cases of acute appendicitis, three of gastroschisis, two cases each of complicated meconium ileus, two with enteric perforations, two congenital diaphragmatic hernia, and one with small bowel atresia. In two patients with acute appendicitis, owing to the inflamed and friable nature of the caecum and terminal ileum site of Meckel’s diverticulum was chosen for ileostomy. The remaining two cases of Meckel's diverticulum were left as such due to a wide base and no palpable abnormality. All cases of complicated meconium ileus underwent ileostomy with simultaneous resection of Meckel’s diverticulum. In case of small bowel atresia, Meckel’s was resected followed by anastomosis. Both cases of enteric perforation required ileostomy and Meckel’s diverticulum was resected. In both cases of congenital diaphragmatic hernia, Meckel’s diverticulum was not excised. Overall, in this study, wedge resection of ileum was performed in 21 cases, 32 cases required segmental resection followed by end-to-end anastomosis, and 23 cases required ileostomy after segmental resection. Due to inadequacy of available record, histopathological results could be retrieved only for 47 (50.50%) specimens, of which ectopic mucosa was found in five (10.63%) cases, whereas the remaining 42 (89.36%) cases showed tissue resembling intestinal mucosa. Ectopic mucosa was confirmed in three patients who presented with per rectal bleeding and in two other cases who presented with abdominal pain due to inflamed Meckel’s diverticulum. Overall, ectopic gastric tissue was found in four cases including three patients with per rectal bleeding and one patient had pancreatic tissue. Mortality was seen in five (5.81%) patients, including two each of meconium ileus and umbilical prolapse of ileal loops, and one with volvulus. The reason for their death was sepsis and electrolyte imbalance.

## Discussion

OMD remnants present as a wide spectrum of anomalies depending on the stage of arrest of normal process of involution, affecting the male population predominantly [8]. Similar sex distribution was observed in the current series. Intestinal obstruction is the most common presentation of OMD remnants with underlying mechanism such as bands, intussusception, or volvulus [9]. In our study, more than half of the patients presented with intestinal obstruction. Meckel’s diverticulum forming bands with surrounding small bowel or the mesentery was the most predominant cause seen in 70.45% of cases. Meckel’s diverticulum can act as a lead point in secondary intussusceptions [10]. This was the second common cause of intestinal obstruction observed in 20% cases. Broad-based Meckel’s diverticulum is more prone to get invaginated into the lumen of the small bowel and drag along, leading to intussusception [11]. In our study, all cases of Meckel’s diverticulum involved in intussusception were found to be of this morphology. Inflamed Meckel’s diverticulum may present with abdominal pain mimicking simple or complicated appendicitis [12,13]. In our study, there were nine cases that were initially diagnosed as acute appendicitis but per-operatively proved to be Meckel’s diverticulitis. Similarly, three patients were assumed as complicated appendicitis but on laparotomy revealed perforated Meckel’s diverticulum. Umbilical anomalies were the second most common manifestation of OMD-related malformations. Because of obvious clinical features, these anomalies are picked early by caregivers or physicians. However, careful assessment is required to differentiate them from a variety of anomalies that can occur at this site such as umbilical granuloma, urachal remnants, or omphalitis [14]. The common presentation of OMD-related anomalies is umbilical discharge, with POMD being the predominant cause, whereas umbilical polyp is rarely seen. Similar are the finding of our study. In our series, the presence of POMD was diagnosed clinically due to gut contents coming out of the umbilical opening. In two cases, however, a contrast study was performed due to uncertain nature of umbilical discharge to differentiate it from other anomalies such as umbilical sinus and patent urachus. Umbilical polyp is a rare remnant of OMD duct and appears as bright red pedunculated growth coming out of the umbilicus and is often mistaken for umbilical granuloma [15,16]. Likewise, in our study, all three cases were initially treated as umbilical granuloma with silver nitrate application. Failure to achieve regression leads to excision, and histopathology results showed intestinal mucosa rather than proliferating capillary network seen in the granuloma. Controversy exists regarding the need for abdominal exploration in case an ectopic umbilical gastrointestinal tissue is discovered to be co-existent with intra-abdominal component of OMD remnant [17,18], In our study, umbilical or abdominal exploration was not performed, and all patients remained asymptomatic on follow-up. We recommend abdominal exploration only if clinically indicated. For example, two patients initially labeled and treated as cases of umbilical granuloma had underlying POMD that eventually required excision after laparotomy. Likewise, exploration was performed and umbilical sinus was excised; however, it did not reveal any intra-abdominal component of OMD remnant. About 2% cases of Meckel’s diverticulum have ectopic mucosa and can lead to ulceration and rectal bleeding [19]. Symptomatic Meckel’s diverticulum should be treated by surgical resection. The choice of surgical techniques depends on its external appearance [9]. In cases of broad-based Meckel’s diverticulum, segmental resection is recommended to avoid restricting the intestinal lumen as well as to ensure full resection of any ectopic gastric tissue and bleeding intestinal ulcer [20,21]. Peptic ulcers resulting from ectopic gastric acid production are often located in the ileum rather than the Meckel’s diverticulum itself due to peristaltic activity in the Meckel's and the resistance of the ectopic gastric tissue to the acid it produces. For long, thin, and non-bleeding Meckel's diverticulum, wedge resection should suffice, as any ectopic tissue within is likely to be confined to the tip [22]. No morbidity was observed in any of the 22 patients who underwent wedge resection in the current series, and this treatment is considered a good option in selected cases with no palpable ectopic tissue. In about 15.16% cases, Meckel’s diverticulum was discovered incidentally. Excision of incidentally discovered Meckel’s diverticulum is a controversial one. Many surgeons advocate that incidentally found normal-appearing Meckel's diverticulum should not to be resected unless there is a palpable abnormality (suggestive of the presence of ectopic mucosa), a long diverticulum (>4 cm), and a narrow neck of diverticulum [23]. In our study, there were cases such as enteric perforation, complicated acute appendicitis, and meconium ileus where ileal diversion was warranted and Meckel’s diverticulum was chosen for ileostomy after its segmental resection. The patient in whom it was left as such had no symptoms at follow-up. This study provides a comprehensive analysis of OMD-related anomalies with regard to their mode of presentation, clinical and surgical findings, and clinical outcome. There are some obvious limitations to our study. Due to the retrospective nature of the study, data were lacking in terms of length of hospital stay, post-operative complication, and long-term follow-up. Histopathology results were also not available for many patient due to inadequacy of medical records at the institute. In addition, due to a wide variability in OMD anomalies, their presentation, surgical management, and outcome, this study could not draw a conclusion to guide the treatment.

## Conclusions

Symptomatic OMD remnants constitute a wide spectrum of anomalies ranging from the umbilicus to their intra-abdominal components. In older children, the predominant presentation is intestinal obstruction, whereas umbilical anomalies are seen in younger children. Surgery is curative and can be tailored as dictated by the type of anomaly. Incidentally discovered Meckel’s diverticulum can be left alone for follow-up or chosen for excision depending on its associated presenting surgical condition.
